# Management of a crown-root fracture: A novel technique with interdisciplinary approach

**DOI:** 10.4317/jced.54811

**Published:** 2018-06-01

**Authors:** Amaia Artieda-Estanga, Pablo Castelo-Baz, Alba Bello-Castro, Isabel Ramos-Barbosa, Benjamin Martin-Biedma, Juan Blanco-Carrion

**Affiliations:** 1 University of Santiago de Compostela, Santiago de Compostela, Spain

## Abstract

Anterior teeth with subgingival fractures require a complex treatment plan that addresses biologic, functional and aesthetic factors. This case report describes the management of a crown-root fractured maxillary left central incisor. An interdisciplinary approach was used to restore the tooth due to the complex nature of the treatment. Orthodontic extrusion was performed to move the fracture line above the alveolar bone and periodontal surgery to recontour the altered gingival margin. Finally, the incisor was restored performing a root canal retreatment with a fiber post and a full ceramic crown. The treatment resulted in secured periodontal health and good aesthetics.

** Key words:**Crown-root fracture, orthodontic extrusion, crown lengthening, root canal retreatment, full ceramic crown.

## Introduction

Crown-root fractures involve enamel, dentine and/or pulp and comprise up to 5% of all traumatic injuries. They are usually caused by direct trauma and maxillary anterior teeth are most often affected due to their anterior and labial relationship with the mandibular incisors.

Invasion of biological width by fracture line presents a clinical challenge in restorative planning (1) and may require a combination of endodontic, periodontal, orthodontic and restorative procedures. Indication of the type of treatment depends on the amount of remaining tooth structure and level of the fracture line (2). The objective in the treatment is to expose sound supragingival tooth structure and make certain that all procedures can be managed with moisture and bleeding control (3).

Extraction, surgical crown lengthening, surgical extrusion and orthodontic extrusion are possible treatment options for a fractured tooth involving the biologic width. Extraction following an implant-supported restoration seems to be the easiest choice, yet it is important to keep in mind that the main advantages of a tooth compared to an implant are the proprioception and the adaptation under mechanical forces, mediated by the periodontal ligament (4). Surgical crown lengthening may result in poor aesthetics and the values of the surgical extrusion on a long-term basis are not yet clear. Orthodontic extrusion is a conservative procedure with good prognosis and does not involve loss of periodontal support or bony tissue of the surrounding teeth (5,6).

In this case report, an interdisciplinary management of a subgingivally fractured tooth assisted by orthodontic extrusion is presented.

## Case Report

**Figure 1:**
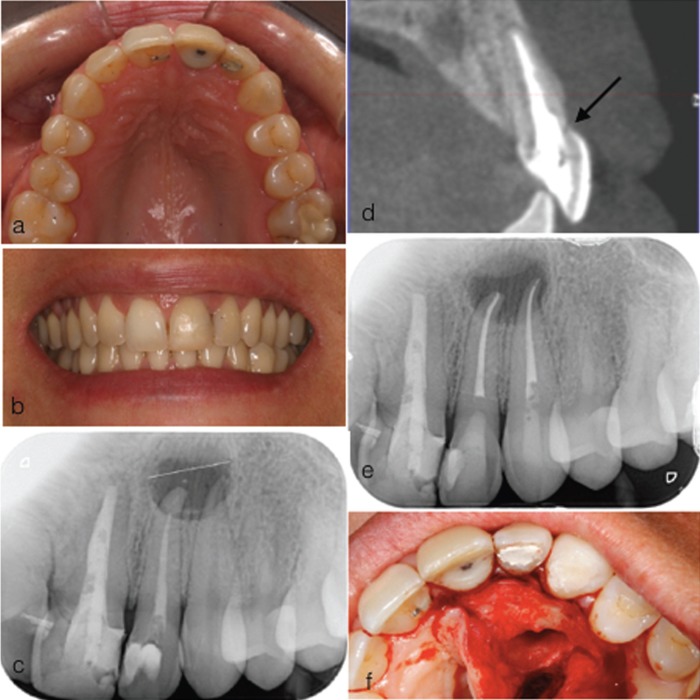
(a) Pre-operative palatal view (b) pre-operative labial view (c) pre-operative radiograph (d) CBCT, arrow showing fracture line (e) root canal treatment of UL2 and UL3 (f) cyst enucleation.

A 42 year-old woman was referred to the Master of Endodontics of the University of Santiago de Compostela with a chief complaint of a subgingivally fractured permanent maxillary left central incisor as a result of a domestic accident. Her medical history was unremarkable. Clinical and radiographic examinations were conducted. Clinical examination revealed a heavily restored maxillary left central incisor that was tender to palpation (Fig. 1A,B) and periapical radiograph and a CBCT revealed an oblique crown-root fracture that extended approximately one-third of the root length (Fig. 1C,D). Radiographic findings showed periapical radiolucencies in the adjacent upper left lateral incisor and canine (Fig. 1C), both teeth remaining negative to cold testing. The diagnosis was a globulomaxillary cyst, both teeth were root canal treated (Fig. 1E) and the cyst enucleated (Fig. 1F).

**Figure 2:**
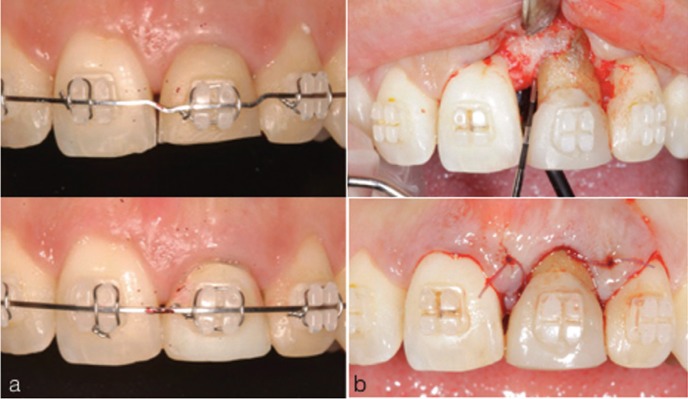
(a) Orthodontic extrusion (b) crown lengthening.

**Figure 3:**
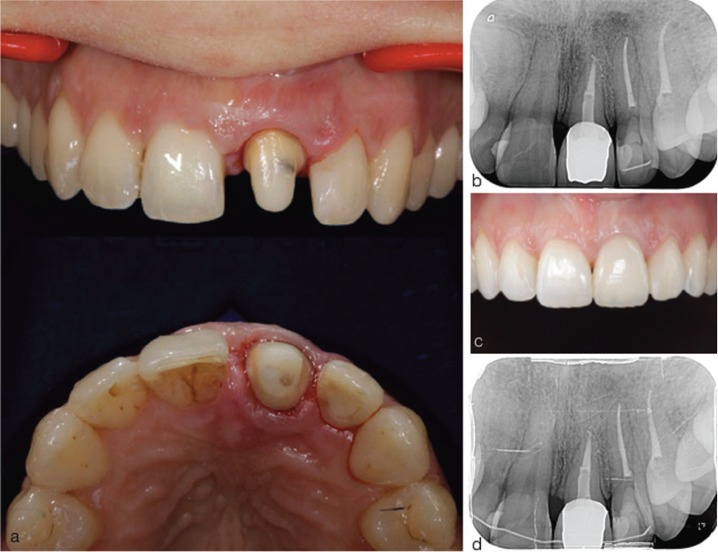
(a) Crown preparation (b) post-operative radiograph (c) post-operative labial view (d) radiograph taken after 4 years of treatment.

In order to regain the lost biologic width, orthodontic extrusion of the fractured permanent maxillary central incisor was required to move the vestibular fracture line approximately 6 mm above the alveolar crest. For the orthodontic extrusion, brackets were attached from upper right first premolar to upper left first premolar. An extrusion of approximately 6 mm was obtained within 6 months (Fig. 2A) and the extruded tooth was retained for 6 months. Periodontal surgery was performed to recontour the altered gingival and osseous margins at the end of the retention period. The root canal retreatment was performed and a fiber post was placed using a dual-cure cement. The post core was built up with a composite and the tooth was prepared for a crown (Fig. 3A). During the time it took to obtain the permanent restoration, the tooth was restored with a temporary crown and an external full mouth tooth whitening was performed before determining the shade of the permanent crown. The ceramic crown was seated to the prepared tooth (Fig. 3B,C) and the upper right central incisor was restored using composite. Good aesthetics were achieved and the patient reported no problems after 4 years of treatment (Fig. 3D). Patient’s informed consent was obtained.

## Discussion

Subgingival crown fractures are challenging in terms of coronal rehabilitation. The location of the fracture line in fractured teeth affects not only the treatment options but also the prognosis of a fractured tooth. Various treatment approaches have been indicated for subgingivally fractured anterior teeth: orthodontic extrusion, surgical extrusion and extraction followed by surgical implants.

A frequent dilemma in dentistry is whether to restore a compromised tooth or to extract it and replace it with an implant-supported restoration. When implants are placed in ideal positions, with adequate prothetic designs and proper maintenance they can achieve high success rates, especially in periodontally healthy patients (7) and non-smoker patients (8), this being the case of our patient. The meta-analysis by Jung and colleagues (9) calculated an implant survival rate of 97.2% after 5 years, decreasing to 95.2% after 10 years.

In this case, the patient was willing to save the tooth and the decision to retain the tooth was taken. Orthodontic extrusion was used to reestablish the biological width and expose the fractured subgingival margins. It is considered to be the easiest tooth movement with a good prognosis and it is defined as an orthodontic movement in which teeth are moved coronally with the application of low intensity and continuous forces with the aim to produce changes on the bone and soft tissues. Based on the literature, we can differentiate between slow orthodontic extrusion and rapid orthodontic extrusion. In the first one, low intensity and continuous forces with periodic orthodontic activations allow reorganisation of PDL fibers in a way that tooth supporting tissues migrate coronally accompaying the extruded tooth. This procedure would be indicated when it is pursued an improvement in oseous and gingival anatomy, for aesthetic reasons or implant placement. On the contrary, stronger traction forces are exerted on the rapid orthodontic extrusion and the activations are more frecuent, coronal migration of the soft tissues being less pronounced due to the lack of PDL fibers reorganisation and bone remodeling. This last technique is indicated for the treatment of subgingival fractures or caries and when there is need for ferrule incrementation, as in the present case (10). However, rapid activations and strong traction forces that do not allow periodontal fiber reorganisation are more associated with gingival inflammation, post-treatment relapse and severe radicular resorptions. Nevertheless, on the literature it is not clearly described which traction forces and activation periodicity are needed for an orthodontic extrusion to be slow or rapid (11). In this case, 1 mm per month were extruded to prevent aforementioned adverse effects.

The root length of the fractured incisor must allow the tooth to undergo the necessary amount of extrusion and still retain a crown-to-root ratio of approximately 1:1 (12). In the present case, the root length of the incisor was enough for the extrusion. It is also important to take into consideration the minimum 2mm ferrule height. Higher ferrule produces more favorable stress distribution between the post-core and core-root in endodontically treated teeth expecting to lower the probability of clinical failure. In this case, a fiber post and a composite core were utilised. Important advantages of fiber posts are the tendency to concentrate stresses along the adhesive interface (13) reducing the risk of tooth fracture and the satisfactory aesthetic appearance with no risk of gingival discoloration (14).

Prior to final restoration the root was retained in its new position for 6 months allowing bone remodelation and periodontal fibers reorganisation to prevent relapse. After this period of time, altered gingival margins were recontoured surgically to obtain aesthetically acceptable soft tissue contours for the posterior prosthetic rehabilitation.

The main disadvantages of this technique are the length of treatment, the need to use orthodontic appliances and poor aesthetics during treatment. This is why it is important for the patient to be fully aware of different treatment options and once the decision has been taken to undergo orthodontic extrusion, both the patient and the dentist require to be committed and motivated.

In this case report, the need for a interdisciplinary approach in the treatment of dental traumas requiring comprehensive treatment has been recognised (15) in respect to biological, functional, and aesthetic aspects.

## Conclusions

This report provides a highly conservative approach that combines function, health of periodontal tissues and aesthetics, postponing the extraction of a compromised anterior incisor.
